# Clinical Characteristics and Survival Outcomes in a Cohort of Pediatric Rhabdomyosarcoma Patients: The Impact of Risk-Adapted Therapy

**DOI:** 10.3390/cancers18111848

**Published:** 2026-06-04

**Authors:** Yanhua Li, Yangyang Jiao, Xuelian Liao, Jingbo Shao, Ting Zhang, Can Huang, Jingwei Yang, Shayi Jiang

**Affiliations:** Department of Hematology and Oncology, Shanghai Children’s Hospital, School of Medicine, Shanghai Jiao Tong University, Shanghai 200040, China; liyh@shchildren.com.cn (Y.L.); jiaoyangyang315@sina.com (Y.J.); xuelianliao@163.com (X.L.); shaojingbo@163.com (J.S.); ymnlzt@163.com (T.Z.); huangcan@shchildren.com.cn (C.H.)

**Keywords:** pediatric rhabdomyosarcoma, risk stratification, treatment outcome, prognostic factor, delayed primary excision

## Abstract

**Background:** Rhabdomyosarcoma (RMS) is the most common pediatric soft tissue sarcoma, with survival dependent on risk stratification and multimodal therapy. This single-center study explored the clinical characteristics, treatment outcomes and prognostic factors of pediatric RMS to optimize local management. **Methods:** Data of RMS patients treated at Shanghai Children’s Hospital from 2011 to 2024 were analyzed to estimate event-free survival (EFS) and overall survival (OS), and factors associated with survival. Patients received the Rs-99 (pre-2019) regimen or a modified Rs-2018 (post-2019) regimen. **Results:** A total of 76 RMS patients were identified. The 5-year EFS and OS for the entire cohort were 71.1% (95% CI, 62.3% to 83.3%) and 72.4% (95% CI, 64.0% to 84.5%), respectively. No statistically significant differences were observed in EFS and OS between the Rs-2018 and Rs-99 regimens. Univariate survival analysis indicated that metastasis was associated with prognosis: the 5-year EFS and OS of patients with metastatic disease were 35.0% (95% CI, 18.3% to 57.6%) and 40.0% (95% CI, 27.3% to 75.3%), while both the 5-year EFS and OS of patients with localized disease reached 83.9% (95% CI, 69.3% to 93.6%). Primary tumor resection status was a key prognostic factor for patients with localized disease, with a 5-year EFS of 100% for R0 resection and 46.7% (95% CI, 25.2% to 74.0%) for R2 resection. For patients with localized disease, EFS was comparable between those who underwent delayed primary excision (DPE) and upfront resection. **Conclusions:** Outcomes for patients with metastatic RMS remain poor. For those with localized disease, primary tumor resection status correlates with improved EFS and OS; additionally, DPE represents a feasible therapeutic option for localized RMS involving complex anatomical sites.

## Simple Summary

Rhabdomyosarcoma is a type of cancer that arises in the muscles and soft tissues, most commonly affecting children. While treatment has improved, outcomes can vary greatly, especially if the cancer has spread. This research was conducted to understand the clinical features, treatment effects, and key prognostic factors of pediatric rhabdomyosarcoma, and to compare two common treatment protocols. We aimed to identify factors that can help improve survival and guide clinical practice. Our study shows that tumor metastasis and complete surgical removal significantly affect patient survival, and delayed surgery is a safe and effective option for tumors in complex locations. These findings can help clinicians optimize risk stratification and multidisciplinary treatment, thereby improving the prognosis of children with this tumor.

## 1. Introduction

Rhabdomyosarcoma (RMS) is the most common soft tissue sarcoma in children and young adults, with an annual incidence of approximately 4.5 cases per million in persons aged less than 20 years [1]. In the United States, 350–500 children develop RMS annually. In some Asian regions, the incidence is lower, with Japan, India and China reporting 2 cases per million persons [2,3]. The disease exhibits a bimodal age distribution, with a younger peak between 2 and 6 years of age and an older peak at 10 to 18 years [1].

The 2020 WHO classification retains the four primary rhabdomyosarcoma subtypes—embryonal (ERMS), alveolar (ARMS), pleomorphic (PRMS), and spindle cell/sclerosing (SRMS)—while also acknowledging several novel variants [4]. Concurrently, the prognostic significance of molecular diagnostics has been amplified. Beyond the established *PAX/FOXO1* fusion genes, markers such as *MYOD1* and *TP53* have been integrated into risk stratification frameworks for the latest Children’s Oncology Group (COG) clinical trials [5].

The risk stratification system established by the Intergroup Rhabdomyosarcoma Study Group (IRSG) is widely used internationally to guide treatment. This system classifies patients into low-risk (LR), intermediate-risk (IR), and high-risk (HR) groups. The application of multimodal therapies, including chemotherapy, surgery and radiotherapy, has improved patient overall survival to 71% [2].

This study retrospectively analyzed 76 cases of RMS treated at Shanghai Children’s Hospital, evaluating treatment outcomes and prognostic factors, with the aim of optimizing risk stratification and comprehensive management to further enhance survival in pediatric patients.

## 2. Materials and Methods

### 2.1. Patient Selection

**Figure 1:**
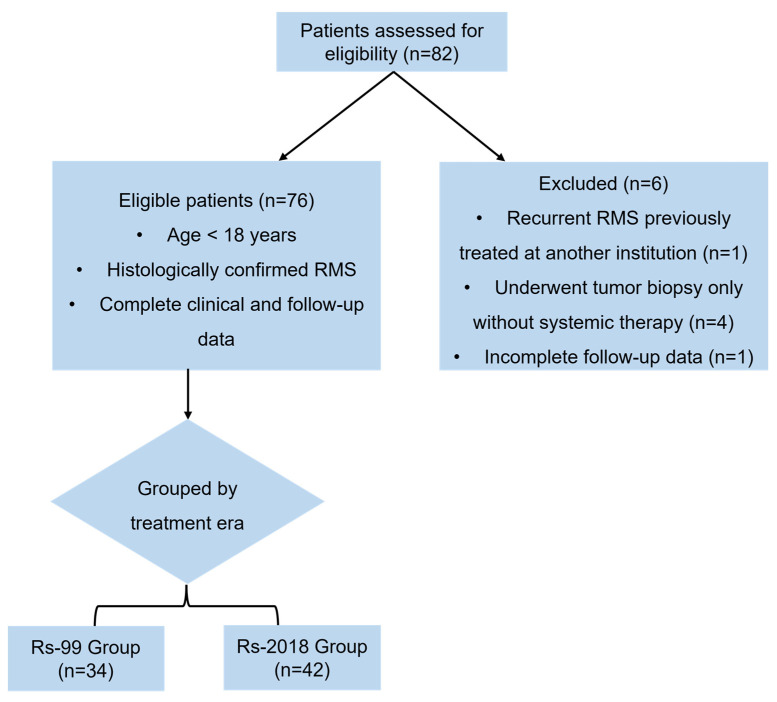
CONSORT diagram for our study.

A total of 76 pediatric patients with RMS treated at our institution between January 2011 and December 2024 were retrospectively reviewed. The inclusion and exclusion criteria are illustrated in the study flowchart (Figure 1). The study was approved by the IRB committee.

### 2.2. Diagnosis

Primary histology was confirmed either by pathologic assessment of the core biopsy or open surgical excision. The pathological diagnosis according to the World Health Organization classification system for RMS includes four subgroups: embryonal rhabdomyosarcoma (ERMS), alveolar rhabdomyosarcoma (ARMS), pleomorphic rhabdomyosarcoma (PRMS), and spindle cell/sclerosing rhabdomyosarcoma (SRMS) [4]. For patients with ARMS after January 2018, *FOXO1* fusion gene testing was performed using fluorescence in situ hybridization. After 2019, all patients were tested. *FOXO1* gene rearrangement was assessed using a dual-color break-apart FISH probe kit (Guangzhou LBP Medicine Science & Technology Co., Ltd., Guangzhou, China) following the manufacturer’s protocol. One hundred tumor cells per sample were evaluated. The normal pattern showed two yellow fusion signals with adjacent red/green signals. *FOXO1* gene rearrangement was identified by a break-apart signal pattern (one yellow, one red, and one green signal). The positivity threshold was set at >15% of cells showing the rearrangement pattern [6]. 

### 2.3. Risk Stratification

Patients enrolled prior to 2019 received treatment according to the Rs-99 protocol, with risk stratification based on the IRS grouping Classification (Table 1) [7]. For patients enrolled after January 2019, the Rs-2018 protocol was used. Risk stratification, which was based on UICC-TNM staging, IRS grouping, and histology, was slightly modified from the criteria of the Children’s Oncology Group (COG) Soft Tissue Sarcoma (STS) Committee’s sixth clinical study (COG-STS-VI) (Table 2) [8,9]. Specifically, ERMS patients with the following stages and groups (Stage 1, Group III and Stage 3, Group I/II) were reassigned to the IR group. The ongoing RMS clinical trial ARST1431 (NCT02567435) conducted by the COG has also categorized these two subsets of patients as IR-RMS [10].

### 2.4. Therapeutic Modalities

The initial treatment included multidrug chemotherapy regimens, radiotherapy and surgery.

#### 2.4.1. Chemotherapy

All patients received chemotherapy according to their risk group, following either the Rs-99 or the Rs-2018 protocol. The Rs-99 regimen involved alternating cycles of DVCP and IVE [4]. Patients in the LR, IR, and HR groups received 6, 12 and 18 cycles of chemotherapy, respectively [4]. The Rs-2018 regimen was administered as follows: the LR group received 9 cycles of the VAC/VA regimen; the IR group received alternating DVAC/IVE followed by VAC/AEV; and the HR group received the IR regimen with the addition of irinotecan and sirolimus (Appendix A). We included patients who were considered to be at high risk of relapse: those with localized incompletely resected ERMS occurring at unfavorable sites with age (≥10 years) or tumor size (>5 cm), or both; those with any localized RMS with regional lymph node involvement; and those with localized ARMS but without regional lymph node involvement. Those in clinical remission after standard treatment received maintenance chemotherapy (six 28-day cycles of intravenous vinorelbine 25 mg/m^2^ on days 1, 8, 15 and daily oral cyclophosphamide 25 mg/m^2^ on days 1–28) [11].

#### 2.4.2. Radiotherapy

All patients received radiotherapy, except those with *FOXO1*-negative rhabdomyosarcoma who achieved complete primary tumor resection at initial surgery [12,13].

#### 2.4.3. Surgery

Except for ARMS, primary tumor resection can be performed upfront on the premise of preserving organ functions, effectively reducing chemotherapy intensity or lowering postoperative radiotherapy doses. The intent of each operation should be the complete excision of disease with negative margins unless such an approach would result in unacceptable loss of function or cosmetic form [14,15].

### 2.5. Follow-Up Assessment

Patients received regular surveillance throughout treatment and follow-up. Tumor response and progression were evaluated using the revised Response Evaluation Criteria in Solid Tumors (RECIST) guideline (version 1.1) [16]. The follow-up cutoff date was 30 June 2025. EFS was defined as the time from diagnosis to disease progression, relapse, second malignancy, or death from any cause. OS was defined as the time from diagnosis to death from any cause.

### 2.6. Statistical Analysis

The Kaplan–Meier method was employed to estimate EFS and OS distributions. Differences between survival curves based on patient, disease, or treatment characteristics were analyzed using the log-rank test. Categorical variables were analyzed using the chi-square test or Fisher’s exact test as appropriate. All the tests were two-tailed with statistical significance defined as *p* < 0.05, and analysis was performed using SPSS 27.0 (released 2020, IBM Corp, Armonk, NY, USA).

## 3. Results

### 3.1. Clinical Characteristics

A total of 76 patients were identified, with a median age at diagnosis of 3.3 years (IQR, 2–6 years) and a 1.2:1 male-to-female ratio. In total, 51 (67.1%) patients were aged <5 years, 19 (25.0%) were aged 5–10 years, and 6 (7.9%) were aged ≥10 years. The five most common primary tumor sites were parameningeal (*n* = 15, 19.7%), trunk and extremity (*n* = 15, 19.7%), head and neck (*n* = 12, 15.8%), bladder/prostate (*n* = 8, 10.5%), and abdominopelvic (*n* = 8, 10.5%). Tumor size was >5 cm in 41 (53.9%) cases. Histologic subtypes were ERMS in 62 (81.6%) patients, ARMS in 11 (14.5%), and SRMS in 3 (3.9%). The *FOXO1* fusion status was available for 34 patients, of whom 7 were fusion-positive, all with ARMS. In total, 56 (73.7%) patients had localized disease and 20 (26.3%) patients had metastatic disease. Among the patients with metastatic disease, bone (35.0%) was the most common site of metastasis, followed by lungs (30.0%). No significant differences were found in baseline clinical characteristics between patients treated with the Rs-2018 and Rs-99 protocols. Clinical characteristics are summarized in Table 3.

### 3.2. Treatment

All patients received systemic chemotherapy. Of these, 42 (55.2%) were treated with the Rs-2018 protocol and 34 (44.8%) with the Rs-99 protocol. Risk stratification yielded 7 (9.2%) patients in the LR group (1 from Rs-99, 6 from Rs-2018), 45 (59.2%) in the IR group (21 from Rs-99, 24 from Rs-2018), and 24 (31.6%) in the HR group (12 from each protocol) (Table 3). Among patients with IRS Group III disease, 12 achieved a complete response (CR) following neoadjuvant chemotherapy, with primary tumors located in the head and neck (*n* = 5), parameningeal region (*n* = 4), orbital region (*n* = 1), vaginal region (*n* = 1), and bladder (*n* = 1).

Among the 56 patients with localized disease, 24 (42.9%) underwent primary tumor resection at initial diagnosis, of whom 21 achieved gross total resection (4 IRS Group I, 17 IRS Group II). Delayed primary excision (DPE) was performed in 23 (41.1%) patients; among these, 6 had negative postoperative pathology, 2 achieved R0 resection, 12 had R1 resection, and 3 had R2 resection. Nine (16.0%) patients did not undergo resection (Table 4).

### 3.3. Survival Outcomes

**Figure 2:**
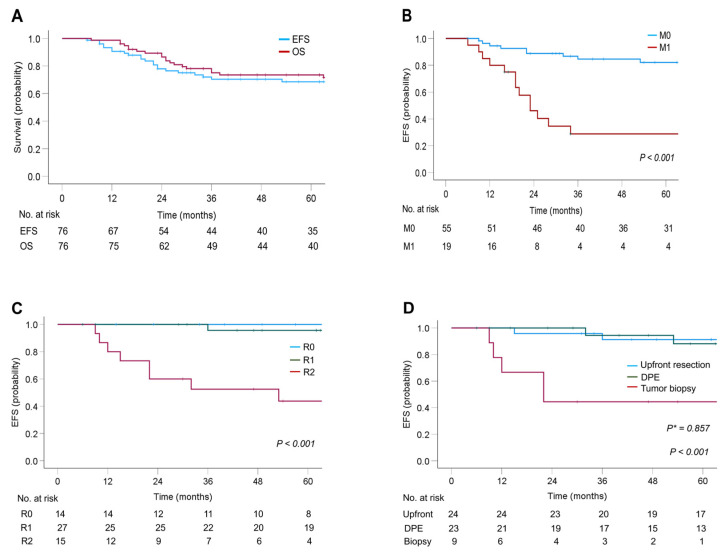
(**A**) The 5-year EFS and OS for the entire cohort. (**B**) EFS by metastatic status. (**C**) EFS by primary tumor resection in patients with localized disease. (**D**) EFS by primary tumor resection time in patients with localized disease; p* represents the comparison between DPE and upfront resection.

The cohort had a median follow-up of 63 months (range, 7–170 months), with a 5-year EFS of 71.1% (95% CI, 62.3% to 83.3%) and a 5-year OS of 72.4% (95% CI, 64.0% to 84.5%) (Figure 2A). For the Rs-2018 and Rs-99 regimens, the 5-year EFS was 73.8% (95% CI, 60.4% to 89.2%) and 67.6% (95% CI, 52.4% to 84.9%), respectively (*p* = 0.68), and the 5-year OS was 76.2% (95% CI, 63.8% to 91.3%) and 67.6% (95% CI, 53.6% to 85.5%), respectively (*p* = 0.80) (Table 5). No significant differences in outcomes were observed between the two treatment regimens in the risk subgroup analysis (Table 6).

Univariable analyses of survival revealed that metastatic disease at diagnosis was the most impactful factor on EFS and OS (Figure 2B). The 5-year EFS and OS of patients with metastatic disease were 35.0% (95% CI, 18.3% to 65.6%) and 40.0% (95% CI, 27.3% to 75.3%), while both the 5-year EFS and OS of patients with localized disease reached 83.9% (95% CI, 69.3% to 93.6%). Age at diagnosis, sex, and tumor size were not statistically significantly associated with EFS and OS.

Moreover, EFS and OS by histologic subtype (ARMS or ERMS) were not statistically significantly different in univariate survival analysis likely because of the low number of ARMS cases in our cohort (11 of 76).

Primary tumor resection was a key prognostic factor for patients with localized disease. The 5-year EFS was 100.0% for R0 resection, 96.3% (95% CI, 72.9% to 99.3%) for R1 resection, and 46.7% (95% CI, 25.2% to 74.0%) for R2 resection (*p* < 0.001; Figure 2C, Table 4). Notably, for patients with localized disease, the 5-year EFS was comparable between those undergoing delayed primary excision (DPE) and those undergoing upfront resection (91.3% vs. 91.7%, *p* = 0.857; Figure 2D; Table 4), supporting the feasibility of DPE as an organ-preserving strategy. In patients with localized disease undergoing DPE, a descriptive analysis of recurrence events across different primary tumor sites is summarized in Table 7. No recurrence events were observed in patients with tumors located in the head and neck (*n* = 11) or extremities (*n* = 3). In contrast, recurrence occurred in 1 out of 4 (25.0%) patients with genitourinary tumors and in 1 out of 5 (20.0%) patients with tumors at other sites. Among the patients with localized disease, 9 cases experienced recurrence, of whom 8 were in postoperative Group 3 and all had the embryonal histologic type.

Of the 20 patients with metastatic disease, 13 (65.0%) experienced disease relapse or progression, with 7 (35.0%) having only metastatic relapse, 3 (15.0%) having local or locoregional relapse, and 3 (15.0%) having both local and metastatic relapse. In the univariable analysis, the Oberlin score was not statistically significant for EFS (55.6% (95% CI, 22.5% to 88.7%) vs. 18.2% (95% CI, 6.3% to 71.6%); Table 8), due to the small sample size.

## 4. Discussion

Rhabdomyosarcoma (RMS) originates from undifferentiated primitive mesenchymal cells with the potential to differentiate into rhabdomyoblasts and can arise in any anatomical site [4,17]. ERMS is the most common subtype, with children less than 5 years of age being most commonly affected. ARMS is recognized to occur in adolescents and young adults [5,18]. Among the 76 pediatric patients enrolled in this study, male patients were predominant. Over half of the patients were under 5 years of age at diagnosis, and the head and neck region was the most frequent primary site [2,3]. These epidemiological and clinical characteristics are largely consistent with data reported by the Children’s Oncology Group (COG) [2,5]. Pathologically, the cohort comprised 62 cases of ERMS, 11 cases of ARMS, 2 cases of spindle cell RMS, and 1 case of sclerosing RMS. The 5-year survival rates showed no significant difference among the various pathological types, a finding that may be influenced by the uneven distribution of case numbers.

According to the latest clinical trial results from the COG, the overall 5-year OS and EFS are 83.7% and 70.8%, respectively [19,20]. In our center, the 5-year EFS was 71.1%, which is consistent with the COG data; however, the 5-year OS was 72.4%, slightly lower than that reported by COG, likely because most children who experienced relapse forewent salvage therapy. Additionally, the Rs-2018 regimen showed a trend towards improved survival in both EFS and OS compared with the Rs-99 regimen, particularly in the high-risk subgroup, although it did not reach statistical significance.

In this study, the risk distribution was 9% in the low-risk group, 59% in the intermediate-risk group, and 32% in the high-risk group. The proportion of the intermediate-risk group was comparable to that reported by COG, whereas the proportion of the high-risk group was significantly higher than the 12% reported by COG [8]. In stratified risk groups, the 5-year EFS and OS for the low-risk group in our center were both 85.7%. Compared with COG data, our OS was slightly lower than their reported 90%, while our EFS was marginally higher than their 78.6%; however, the gap was not significant [21,22]. For intermediate-risk patients, the 5-year EFS and OS were both 82.2%, better than the outcomes of the COG ARST0531 study (OS 73%, EFS 63%) [23]. This survival advantage may be attributed to our broader inclusion criteria for the intermediate-risk group, which encompassed some patients who would be classified as low-risk under COG standards. These patients achieved a 5-year EFS and OS of 100%. After excluding these individuals, the 5-year EFS and OS of the intermediate-risk cohort dropped to 69.2%. Notably, the survival outcomes of this specific subgroup exceeded the low-risk cohort in the COG ARST0331 study and our own low-risk group, suggesting that these patients may derive greater benefit from intermediate-intensity treatment regimens [21,22]. It is worth noting that the ongoing COG ARST1431 study has similarly reclassified such patients into the intermediate-risk group for treatment [10]. For high-risk patients, the 5-year EFS was 45.8% and OS was 50.0%, figures comparable to those in COG reports [24]. Nevertheless, the prognosis remains unsatisfactory.

The TNM staging reflects the extent of tumor invasion and metastasis, which is closely correlated with prognosis. Patients with stage 4 disease, with the exception of those younger than 10 years who have ERMS, have a poor prognosis [24,25,26]. Neither intensification of chemotherapy by increasing the dose of cyclophosphamide and adding active agents to standard vincristine/actinomycin/cyclophosphamide (VAC) therapy nor use of high-dose chemotherapy with stem-cell rescue has improved outcomes over the past 30 years [24,26,27]. In our center, the 5-year EFS for patients with metastatic disease remains poor at 35.0%. Although an Oberlin score of 1 was associated with better OS, this analysis was limited by a small sample size [24].

Primary tumor resection is a key prognostic factor in patients with localized disease. The data show that the 5-year EFS for patients undergoing R0 resection was as high as 100%, significantly better than the 46.7% for those with R2 resection, underscoring the importance of complete excision [28]. Timing of the primary tumor resection is also crucial. For tumors in complex anatomical locations or those difficult to resect completely upfront, the DPE strategy offers a better balance between tumor control and functional preservation [29]. In our study, the EFS and OS of patients undergoing DPE were comparable to those of patients undergoing upfront tumor resection. Due to the limited sample size in each anatomical subgroup, statistical testing was not performed. Instead, a descriptive analysis of the data revealed distinct trends: DPE appeared to offer a favorable trend for tumor control in extremity tumors. In contrast, the benefits were less pronounced in genitourinary lesions. This anatomical specificity of its advantages has been reported by the COG [29,30,31]. Furthermore, we observed that some patients with head and neck RMS demonstrated high sensitivity to chemotherapy and radiotherapy, and these patients achieved complete tumor remission and long-term survival following neoadjuvant chemotherapy combined with radiotherapy [32]. For such patients, radical surgery may not be mandatory. Several limitations of this analysis warrant consideration. First, this was a single-center, retrospective analysis with a limited sample size. The number of cases in some subgroups was particularly small, which may limit the statistical power of the analysis. Moreover, the extended study period coincided with major advances in comprehensive care, such as more precise imaging, improved radiotherapy, and better supportive treatment. Consequently, the improved survival rates observed may reflect these broader medical advancements rather than the effects of the Rs protocols alone. Lastly, the absence of fusion gene status data for patients treated in the earlier years may have compromised the accuracy of risk stratification, introducing potential classification bias [5,33,34].

## 5. Conclusions

In conclusion, although therapeutic strategies for rhabdomyosarcoma continue to be optimized, patient prognosis still varies substantially. The 5-year survival rate for metastatic disease remains below 50%. Therefore, identifying the prognostic factors is particularly important. The prognosis for the high-risk group remains suboptimal, necessitating the exploration of novel therapeutic approaches.

## Figures and Tables

**Table 1:** Risk stratification used for the Rs-99 protocol.

Risk Group	Histologic Type	IRS Group
LR group	non-alveolar RMS	I
IR group	alveolar RMS	I
	all RMS	II
	non-alveolar RMS	III
HR group	alveolar RMS	III
	all RMS	IV

Abbreviations: RMS, rhabdomyosarcoma; IRS, Intergroup Rhabdomyosarcoma, LR, low-risk; IR, intermediate-risk; HR, high-risk.

**Table 2:** Risk stratification used for the Rs-2018 protocol.

Risk Group	Histologic Type	Stage	IRS Group
LR group	non-alveolar RMS	1, 2	I, II
IR group	non-alveolar RMS	1, 2, 3	III
	non-alveolar RMS	3	I, II
	alveolar RMS	1, 2	I, II
HR group	non-alveolar RMS	4	IV
	alveolar RMS	1, 2, 3, 4	III, IV

Abbreviations: LR, low-risk; IR, intermediate-risk; HR, high-risk.

**Table 3:** Baseline clinical characteristics of all patients and subgroups treated with Rs-99 and Rs-2018 protocols.

	All Patients (*n* = 76)	Rs-99 (*n* = 34)	Rs-2018 (*n* = 42)	
Variable	*n*. (%)	*n*. (%)	*n*. (%)	*p*
**Age (years)**				0.720
<5 years old	51 (67.1)	24 (70.6)	27 (64.3)	
5–10 years old	19 (25.0)	7 (20.6)	12 (28.6)	
≥10 years old	6 (7.9)	3 (8.8)	3 (7.1)	
**Sex**				0.188
Male	41 (53.9)	15 (44.1)	26 (61.9)	
Female	35 (46.1)	19 (55.9)	16 (38.1)	
**Primary site**				0.238
Parameningeal	15 (19.7)	5 (15.6)	10 (23.8)	
Head and neck	12 (15.8)	7 (21.9)	5 (11.9)	
Orbital	5 (6.6)	0	5 (11.9)	
Extremity/Trunk	15 (19.7)	6 (18.8)	9 (21.4)	
Retroperitoneal/perineal/other	17 (22.4)	9 (28.1)	6 (14.3)	
Genitourinary, nonbladder or nonprostate	4 (5.3)	1 (3.1)	3 (7.1)	
bladder/prostate	8 (10.5)	4 (12.5)	4 (9.5)	
**Histology**				0.733
ERMS	62 (81.6)	27 (79.4)	35 (83.3)	
ARMS	11 (15.5)	5 (14.7)	6 (14.3)	
others	3 (3.9)	2 (5.9)	1 (2.4)	
**Maximum tumor size, cm**				1.000
≤5 cm	35 (46.1)	16 (47.1)	19 (45.2)	
>5 cm	41 (53.9)	18 (52.9)	23 (54.8)	
**Metastatic status**				0.307
M0	56 (73.4)	23 (67.6)	33 (78.6)	
M1	20 (26.3)	11 (32.4)	9 (21.4)	
**IRS group**				0.607
I	4 (5.3)	1 (2.9)	3 (7.1)	
II	17 (22.4)	8 (23.5)	9 (21.4)	
III	35 (46.1)	14 (41.2)	21 (50.0)	
IV	20 (26.3)	11 (32.4)	9 (21.4)	
**Risk group**				0.227
Low	7 (9.2)	1 (2.9)	6 (14.3)	
Intermediate	45 (59.2)	21 (61.8)	24 (57.1)	
High	24 (31.6)	12 (35.3)	12 (28.6)	
**RT**				0.686
Yes	70 (92.1)	32 (94.1)	38 (90.5)	
No	6 (0.8)	2 (5.9)	4 (9.5)	

Abbreviations: ERMS, embryonal RMS; ARMS, alveolar RMS; M1, the presence of metastasis; M0, no metastasis; RT, radiotherapy.

**Table 4:** Univariable analysis of factors associated with the 5-year EFS and OS of patients with localized disease (*n* = 56).

Variable	*n*. (%)	5-Year EFS	95% CI	*p*	5-Year OS	95% CI	*p*
**Primary tumor resection**				<0.001			<0.001
R0	14 (25.0)	100			100		
R1	27 (48.2)	96.3	72.9–99.3		96.3	69.5–99.3	
R2	15 (26.8)	46.7	25.2–74.0		46.7	25.2–74.0	
**Primary tumor resection time**				<0.001			<0.001
Upfront resection	24 (42.9)	91.7	73.9–92.3		91.7	73.9–92.3	
DPE	23 (41.1)	91.3	66.6–89.7		91.3	69.5–99.3	
Only tumor biopsy	9 (16.0)	44.4	13.5–71.8		44.4	21.4–92.2	

Abbreviations: R0, complete tumor removal with negative margins; R1, microscopically positive surgical margins; R2, macroscopic residual disease; DPE, delayed primary excision.Radiotherapy was administered to all patients at the primary and metastatic sites, except for six ERMS patients, including two patients who achieved satisfactory local control with surgery alone, two patients because of parental refusal, and two patients who progressed through therapy.

**Table 5:** Univariable analysis of factors associated with the 5-year EFS and OS of patients with RMS (*n* = 76).

Variable	5-Year EFS	95% CI	*p*	5-Year OS	95% CI	*p*
**Total**	71.1	62.3–83.3		72.4	64.0–84.5	
**Trials**			0.675			0.798
Rs-99	67.6	52.4–84.9		67.6	53.6–85.5	
Rs-2018	73.8	60.4–89.2		76.2	63.8–91.3	
**Age, years**			0.245			0.179
<5	76.5	66.6–90.2		78.4	71.8–93.3	
5–10	57.9	38.2–87.7		57.9	39.1–88.4	
≥10	66.7	37.9–94.8		66.7	58.3–95.6	
**Sex**			0.099			0.146
Male	63.4	49.5–80.8		65.9	55.0–85.1	
Female	80	69.3–96.1		80	69.9–96.1	
**Primary site**			0.689			0.66
Parameningeal	66.7	42.2–90.2		66.7	42.2–91.2	
Head and neck	83.3	61.7–99.3		83.3	61.7–99.4	
Orbital	100			100		
Extremity/Trunk	60	34.5–85.5		66.7	43.2–90.2	
Retroperitoneal/perineal/other	70.6	47.1–94.1		70.6	47.1–94.1	
Genitourinary, nonbladder or nonprostate	75	32.5–98.6		75	32.5–98.6	
bladder/prostate	62.5	25.3–99.8		62.5	27.2–97.8	
**Histology**			0.547			0.588
ERMS	69.4	57.9–83.0		71	59.8–83.6	
ARMS	72.7	36.8–94.5		72.7	38.1–94.3	
others	100			100		
**Maximum tumor size, cm**			0.485			0.263
≤5	74.3	62.4–91.9		77.1	62.5–92.2	
>5	68.3	55.6–85.3		68.3	57.1–86.9	
**Metastatic status**			<0.001			<0.001
M0	83.9	69.3–93.6		83.9	69.3–93.6	
M1	35	18.3–65.6		40	27.3–75.3	
**IRS group**			<0.001			<0.001
I	100			100		
II	94.1	63.2–99.1		94.1	78.4–99.8	
III	77.1	62.6–91.6		77.1	63.2–91.0	
IV	35	12.3–57.7		40	17.1–62.9	
**Risk group**			0.001			0.003
Low	85.7	27.1–97.4		85.7	27.1–99.4	
Intermediate	82.2	72.7–95.4		82.2	73.5–95.6	
High	45.8	29.7–72.8		50	32.0–76.2	

Abbreviations: CI, confidence interval; EFS, event-free survival; OS, overall survival; ERMS, embryonal RMS; ARMS, alveolar RMS; M1, the presence of metastasis; M0, no metastasis.

**Table 6:** EFS and OS Rates by Treatment Arm.

Risk Group	EFS			OS		
	Rs-99	Rs-2018	*p*	Rs-99	Rs-2018	*p*
LR	100	83.3 (48.2–99.0)	0.655	100	83.3 (13.4–99.9)	0.480
IR	85.7 (72.6–98.8)	79.2 (63.5–94.9)	0.404	85.7 (70.8–99.0)	79.2 (63.5–94.9)	0.243
HR	33.3 (6.6–60.0)	58.3 (28.1–88.5)	0.208	33.3 (6.6–60.0)	66.7 (38.3–95.1)	0.249

Abbreviations: LR, low-risk; IR, intermediate-risk; HR, high-risk.

**Table 7:** Descriptive analysis of recurrence events across different primary tumor sites in patients with localized disease undergoing DPE.

Primary Tumor Site	*n*. (%)	Recurrence (*n*)	Recurrence Rate (%)
Total	23	2	8.7%
Head and Neck (including parameningeal)	11 (47.8)	0	
Extremities	3 (13)	0	
Genitourinary (including Bladder)	4 (17.4)	1	25.0%
Other Sites	5 (21.7)	1	20.0%

Abbreviations: DPE, delayed primary excision.

**Table 8:** Univariable analysis of factors associated with the 5-year EFS and OS of patients with metastatic disease (*n* = 20).

Oberlin Score	*n*. (%)	5-Year EFS	95% CI	*p*	5-Year OS	95% CI	*p*
≤1 Risk Factor	9 (45.0)	55.6	22.5–88.7	0.055	55.6	22.1–89.1	0.097
≥2 Risk Factors	11 (55.0)	18.2	6.3–71.6		27.3	12.3–78.9	

Abbreviations: Oberlin risk factor, age ≥ 10 years or <1 year at diagnosis, unfavorable site, bone or bone marrow involvement, and three or more metastatic sites.

## Data Availability

The data presented in this study are available on request from the corresponding authors.

## Abbreviations

The following abbreviations are used in this manuscript:

RMS	Rhabdomyosarcoma
DPE	Delayed primary excision
IRSG	Intergroup Rhabdomyosarcoma Study Group
COG	Children’s Oncology Group
EFS	Event-free survival
OS	Overall survival
RECIST	Response Evaluation Criteria in Solid Tumors
ERMS	Embryonal rhabdomyosarcoma
ARMS	Alveolar rhabdomyosarcoma
PRMS	Pleomorphic rhabdomyosarcoma
SRMS	Spindle cell/sclerosing rhabdomyosarcoma
LR	Low-risk
IR	Intermediate-risk
HR	High-risk
CI	Confidence interval

## Appendix A

**Table A1:** Treatment Schema and Chemotherapy Doses.

Week	Treatment				Chemotherapy Doses
	Rs-99	Rs-2018			
		LR	IR	HR	
0					
1	DV*CP	VAC*	DVAC	D*VAC	V*: vincristine 1.5 mg/m^2^/d1,8
2					V: vincristine 1.5 mg/m^2^/d1,8,15 (max dose 2 mg)
3					D: adriamycin 30 mg/m^2^/d2,9
4	IV*E	VAC*	IVE	I*VE	D*: adriamycin 35 mg/m^2^/d2,9
5					C: cyclophosphamide 300 mg/m^2^/d × 3
6					C*: cyclophosphamide 1.200 mg/m^2^/d1
7	DV*CP	VAC*	DVAC	V Irin	P: cisplatin 90 mg/m^2^/d1
8					I: ifosfamide 1.5 g/m^2^/d × 5
9	EVAL	EVAL	EVAL	EVAL	I*: ifosfamide 1.8 g/m^2^/d × 5
10	IV*E	VAC*	IVE	D*VAC	E: etoposide 100 mg/m^2^/d × 5
11					E*: etoposide 100 mg/m^2^/d × 3
12					A*: dactinomycin 0.012 mg/kg/d × 5
13	DV*CP	VA	DVAC	I*VE	A: 0.045 mg/kg/d1 (max dose 2.5 mg)
14					Irin: 50 mg/m^2^/d × 5 (max dose 100 mg/d)
15					Sirolimus: 0.1 mg/kg (Rs-2018 HR group)
16	IV*E	VA	IVE	V Irin	
17	EVAL	EVAL	EVAL	EVAL	
18	RT	RT	RT	RT	
19	V*CP	VA	VAC*	VAC*	
20					
21					
22	A*E*V*	VA	AE*V	AEV	
23					
24					
25	V*CP	VA	VAC*	VAC*	
26					
27	EVAL	EVAL	EVAL	EVAL	
28	A*E*V*		AE*V	AEV	
29					
30					
31	V*CP		VAC*	VAC*	
32					
33					
34	A*E*V*		AE*V	AEV	
35					
36	EVAL		EVAL	EVAL	
37	V*CP				
38					
39					
40	A*E*V*				
41					
42					
43	V*CP				
44					
45					
46	A*E*V*				
47					
48					
49	V*CP				
50					
51					
52	A*E*V*				
53					
54	EVAL				
